# Gestational age at delivery of twins and perinatal outcomes: a cohort study in Aberdeen, Scotland.

**DOI:** 10.12688/wellcomeopenres.15211.2

**Published:** 2019-07-22

**Authors:** Sarah R. Murray, Sohinee Bhattacharya, Sarah J. Stock, Jill P. Pell, Jane E. Norman

**Affiliations:** 1 MRC Centre for Reproductive Health, University of Edinburgh Queen's Medical Research Institute, Edinburgh, EH16 4TJ, UK; 2 Institute of Applied Health Sciences, University of Aberdeen, Aberdeen, AB25 2ZL, UK; 3 Institute of Health and Wellbeing, University of Glasgow, Glasgow, G12 8RZ, UK

**Keywords:** Labour, Labour induction, Prematurity, Preterm labour, IVF

## Abstract

**Background: **Twin pregnancy is associated with a threefold increase in perinatal death compared to singletons.  The objective of this study was to determine the risk of perinatal death in twins by week of gestation and to quantify the effect of known risk factors.

**Methods: **A cohort analysis was performed using data from the Aberdeen Maternity and Neonatal Databank (AMND).  The exposure was gestational age at delivery and the primary outcome was perinatal death.  Adjusted hazard ratios (aHRs) for perinatal death according to gestational age at delivery were determined by multivariate Cox proportional hazards regression modelling with robust standard errors to account for clustering in the twin infants.  Confounders and risk factors quantified and adjusted for in the model included maternal age, smoking, parity, marital status and year of birth.  Kaplan-Meier time to event analysis was used to determine the differences in survival according to chorionicity and assisted reproduction technologies (ART) conception status.

**Results:** The population comprised of 7,420 twin babies born between 1950 and 2013 in the Grampian area of Northern Scotland.  There were 272 stillbirths in the cohort (3.67%) and 273 neonatal deaths (3.68%). Compared to delivery at 37-38 weeks, delivery before 37 weeks was associated with a 2-fold increase in perinatal death. Monochorionic twins had a 2-fold increase in perinatal death compared to dichorionic twins (aHR 2.15, 95% CI 1.60-2.90). Twins conceived by ART did not have a greater risk of perinatal death compared to those naturally conceived (aHR 1.21, 95% CI 0.87-1.68)

**Conclusion:  **This study suggests that delivery of twins at 37-38 weeks is associated with the lowest risk of perinatal death.

## Introduction

Twin pregnancies have a threefold greater perinatal death rate overall compared to singleton pregnancies ^1^. The larger perinatal mortality is thought to be due to the greater preterm birth rates in twins with approximately 50% of twins delivering prematurely (less than 37 weeks gestation) compared to 6% of singletons ^2^. The gestation with the lowest absolute perinatal death rate is earlier in twins compared to singletons ^3^. However, delivery before term in singletons has been shown to be associated with an increased risk of neonatal morbidity. Hence risks of perinatal and neonatal mortality and morbidity have to be balanced when making decisions regarding timing of delivery of twins ^4^. 

Despite accounting for only 3% of all live births, multiple pregnancies have a threefold higher economic burden on healthcare systems compared to singleton pregnancies because of the increased caesarean sections and neonatal unit admissions ^5^. Due to increases in assisted reproduction technologies (ART) in recent years the twin birth rate has risen and is set to continue to rise ^6^.

Optimising the timing of delivery is a key strategy in reducing perinatal death and morbidity in twin pregnancies. UK clinical guidelines support a policy of elective delivery from 37 weeks in dichorionic pregnancies (two placentae and two separate chorions) and 36 weeks in monochorionic pregnancies (one placenta and either one or two chorions) ^6^ in order to reduce adverse short term outcomes in twins such as perinatal mortality. This strategy is informed by epidemiological studies on gestational age specific outcomes: however, these studies lack detail about the accuracy of the pregnancy dating, fail to adjust for the clustered outcomes of twin pregnancies and lack information on chorionicity, which is a key risk factor for adverse pregnancy outcome. Monochorionic twins have a perinatal mortality rate of 11.6% compared to 5% in dichorionic twins ^7^. Chorionicity is therefore a very important factor to consider when attempting to determine optimum timing of delivery of twins.

Randomised controlled trials investigating optimum timing of delivery in twins have not been adequately powered to assess perinatal death ^8, 9^. A recent systematic review using data prospectively collected from randomised controlled trials, and therefore different from observational studies, had findings in line with the current UK practice recommending elective delivery from 37 weeks in dichorionic and 36 weeks in monochorionic twins to minimise perinatal deaths ^10^. This review lacked information on whether the twins were conceived by ART procedures or were naturally conceived. This is important because ART pregnancies (twins and singletons) often have additional obstetric risk factors such as advanced maternal age and nulliparity ^11, 12^. Despite good evidence that singleton pregnancies conceived by ART procedures are at increased risk of obstetric and perinatal complications ^13^, the evidence on pregnancy outcomes of twins conceived by ART procedures is conflicting. A systematic review and meta-analysis demonstrated no differences in perinatal outcomes between twins conceived by ART and those naturally conceived ^14^, however some studies showed increased rates of caesarean section and small for gestational age in the twins conceived by ART procedures compared with naturally conceived twin pregnancies ^15^.

This aim of this study was to explore the relationship between gestation at delivery and perinatal death in twins and determine whether this varies by chorionicity and ART conception status.

## Methods

### Study design and participants

We carried out a registry-based cohort study using all twin births in the Grampian area of Scotland between 1950 and 2013. Data were obtained from the Aberdeen Maternity and Neonatal Databank (AMND). The AMND has collected information on pregnancy related events in women living in Grampian since 1950; a relatively stable population with approximately 5,000 births per year. The Aberdeen Maternity Hospital (AMH) is the only maternity facility in Aberdeen city and greater than 99% of residents deliver there ^16^. The database is subject to regular quality assurance checks and completeness of the database is checked annually against the National Health Service (NHS) records. The methods used for data coding (using ICD-10 ^17^ of the AMND and full details of the database have been described previously ^18– 21^. The study was approved by the AMND steering committee (approval number AMND 001/16). Individual patient consent and further ethical approval was not required as the study used secondary analyses of anonymised data. 

### Inclusion and exclusion criteria

Women were included if they had a twin delivery at 24 weeks’ gestation or greater within the study period. Pregnancies complicated by congenital anomaly were excluded. Pregnancies were excluded if the gestational age at delivery was missing or recorded as greater than 43 weeks gestation, maternal age less than 10 years and if parity was missing or recorded as greater than 14.

### Outcomes, exposures and covariates

The exposure of interest was gestational age at delivery. In the AMND this is recorded as the number of completed weeks of gestation on the basis of the estimated date of delivery recorded in the clinical record. Prior to 1985 this was calculated from the date of the last menstrual period with ultrasound scan dating thereafter. Gestational age was treated as an ordinal variable grouped into two-week periods below 34 weeks gestation and one week from 34 weeks. The primary outcome was extended perinatal death of one or both twins defined as either antepartum/intrapartum stillbirth (infant born showing no signs of life) or neonatal death (death of a liveborn infant in the first four weeks of life). For the multivariate analyses and the analyses stratified by chorionicity (binary variable; monochorionic and dichorionic) and ART conception (binary variable; assisted conception/no assisted conception) we further categorised gestational age into the following categories due to the sparsity of events in some of the categories; less than 32 weeks, 33–36 weeks, 37–38 weeks [reference category] and greater than 38 weeks.

The following variables were considered to be potential confounders in the multivariate regression analyses: maternal age at delivery (categorised as <20, 21–24, 25–29, 30–34, 35–39, and >40 years), parity during the index pregnancy (binary variable categorised as para 0 or para ≥ 1), year of birth (categorised as 1950–75, 1976–2000, 2000–2013), area socioeconomic deprivation quintile of postcode of residence (defined by the Scottish Index of Multiple Deprivation [SIMD] 2012; 1 [most affluent] to 5 [most deprived] ^22^), maternal height (categorised as 141–150, 151–160, 161–170, and >170 cm), smoking (categorised as current smoker, ex-smoker or never-smoker), marital status (binary variable categorised as married/co-habiting or single), medically indicated induction of labour and maternal complications in pregnancy (binary variable categorised as no maternal complication or any of pre-eclampsia, hypertensive disease, diabetes or antepartum haemorrhage). 

### Statistical analyses

Summary statistics were derived and compared by gestational age using chi squared test for categorical data and chi-squared test for trend for ordinal data. To determine the association between gestational week of delivery and the risk of perinatal death Cox proportional hazard regression modelling was used. To obtain the adjusted hazard ratios (aHR) for the effect of gestational age at delivery on perinatal death a Cox regression model was fitted with the following covariates – maternal age at delivery, maternal parity, marital status, maternal height and maternal complications. We calculated robust standard errors to account for the clustering of twins within mothers. 

Entries which had missing values were examined in the summary statistics but excluded from the univariate and multivariate analyses. Maternal smoking was not included in the final model due to the amount of missing values but a sensitivity analysis of complete cases with missing cases was performed.

In the subgroups of pregnancies with chorionicity and ART data available the analysis was repeated, stratifying by each variable and the relationship to perinatal death assessed using Kaplan-Meier analyses and Cox proportional hazards models in which gestational age was the time scale and perinatal death the event ^23^. aHRs were estimated, and time-to-event curves compared using the log-rank test. 

P values for hypothesis tests were two sided and statistical significance set at P<0.05. All analyses were undertaken using STATA MP, version 14.1 (stata corporation).

## Results

**Figure 1.**
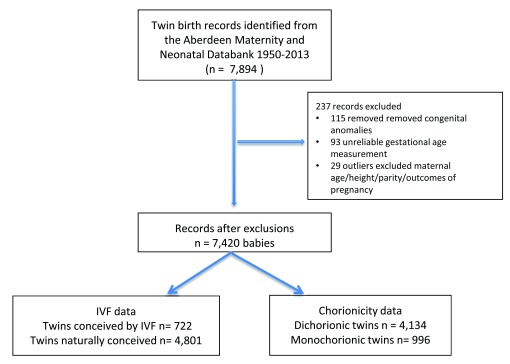
Cohort composition.

The AMND contained 7,894 records of twin infants born in Grampian over the study period of 1950–2013 of which 7,420 were eligible for inclusion in the analysis. There were 544 perinatal deaths (232 stillbirths and 312 neonatal deaths) in this cohort. Figure 1 displays the process of deriving the study cohort.

Table 1 summarises the pregnancy characteristics of the cohort. Among the twin infants, the largest proportion were delivered between 37 and 38 weeks (n = 2,363, 31.83%) and overall 3,615 (48.72%) delivered prematurely (less than 37 weeks gestation).

**Table 1.** Baseline summary statistics of the population of 7,420 twins born in Grampian, Scotland.

Pregnancy Characteristic	Total N	N (%) in each gestation age group in weeks				P value
		24–32	33–36	37–38	≥39	
Perinatal death (combined stillbirth and NND ^a^) No Yes	6876 588	464 (6.8) 276 (50.7)	2774 (40.3) 101 (8.6)	2299 (33.4) 64 (11.8)	1339 (19.5) 103 (18.9)	<0.001
Maternal age 15–20 21–24 25–29 30–34 35–39 >40 Missing	486 1290 2272 2108 1066 198 0	56 (11.5) 170 (13.2) 216 (9.5) 196 (9.3) 80 (7.5) 22 (11.1)	192 (39.5) 513 (39.8) 806 (35.5) 812 (38.5) 458 (43.0) 94 (47.5)	112 (23.1) 345 (26.7) 786 (34.6) 704 (33.4) 366 (34.3) 50 (25.3)	126 (25.9) 262 (20.3) 464 (20.4) 396 (18.8) 162 (15.2) 32 (16.2)	<0.001
Maternal smoking Ex-smoker Smoker Non-smoker Missing	274 1675 3476 1995	34 (12.4) 192 (11.5) 310 (8.9) 204 (10.2)	128 (46.7) 640 (38.2) 1486 (42.8) 621 (31.1)	94 (34.3) 473 (28.2) 1180 (34.0) 616 (30.9)	18 (6.6) 370 (22.1) 500 (14.4) 54 (27.8)	<0.001
Maternal height 141–150 151–160 161–170 >170 Missing	224 2706 3546 702 242	26 (11.6) 300 (11.1) 332 (9.4) 36 (5.1) 46 (19)	98 (43.8) 981 (36.3) 1408 (39.7) 274 (39.0) 114 (47)	64 (28.6) 809 (29.9) 1156 (32.6) 286 (40.7) 48 (20)	36 (16.1) 616 (22.8) 650 (18.3) 106 (15.1) 34 (14)	<0.001
Year of birth 1950–75 1976–2000 2001–2013 Missing	1957 3347 2116 0	168 (8.6) 368 (11.0) 204 (9.6)	577 (29.5) 1142 (34.1) 1156 (54.6)	524 (26.8) 1229 (36.7) 610 (28.8)	688 (35.2) 608 (18.2) 146 (6.9)	<0.001
Parity Primigravida Parous Missing	3077 4343 0	418 (13.6) 322 (7.4)	1335 (43.3) 1540 (35.5)	856 (27.8) 1507 (34.7)	468 (15.2) 974 (22.4)	<0.001
Marital status Married Other ^b^ Missing	6034 1382 4	592 (9.8) 148 (10.7) 0	2231 (37.0) 642 (46.5) 2 (50)	1961 (32.5) 402 (29.1) 0	1250 (20.7) 190 (13.8) 2 (50)	<0.001
Maternal preconditions No Yes Missing	4337 3042 0	506 (1.6) 234 (7.7)	1599 (36.5) 1276 (41.9)	1366 (31.2) 997 (32.8)	906 (20.7) 536 (17.6)	<0.001
Chorionicity Monochorionic Dichorionic Missing	996 4134 2290	112 (11.2) 344 (8.3) 284 (12.4)	506 (50.8) 1658 (40.1) 711 (31.0)	270 (27.1) 1500 (36.3) 593 (25.9)	108 (10.8) 632 (15.3) 702 (30.7)	<0.001
Social deprivation category 1 2 3 4 5 Missing	656 1032 2130 1330 478 1794	66 (10.1) 100 (9.7) 214 (10.1) 112 (8.4) 64 (13.4) 184 (10.3)	268 (40.9) 386 (37.4) 797 (37.4) 538 (40.5) 184 (38.5) 702 (39.1)	194 (29.6) 364 (35.3) 717 (33.7) 418 (31.4) 140 (29.3) 530 (29.5)	128 (19.5) 182 (17.6) 402 (18.9) 262 (19.7) 90 (18.8) 378 (21.1)	0.02

*^a^NND = Neonatal Death*

*^b^Other = (divorced/single/co-habiting/widow)*

### Perinatal outcomes according to gestation at delivery

**Figure 2.**
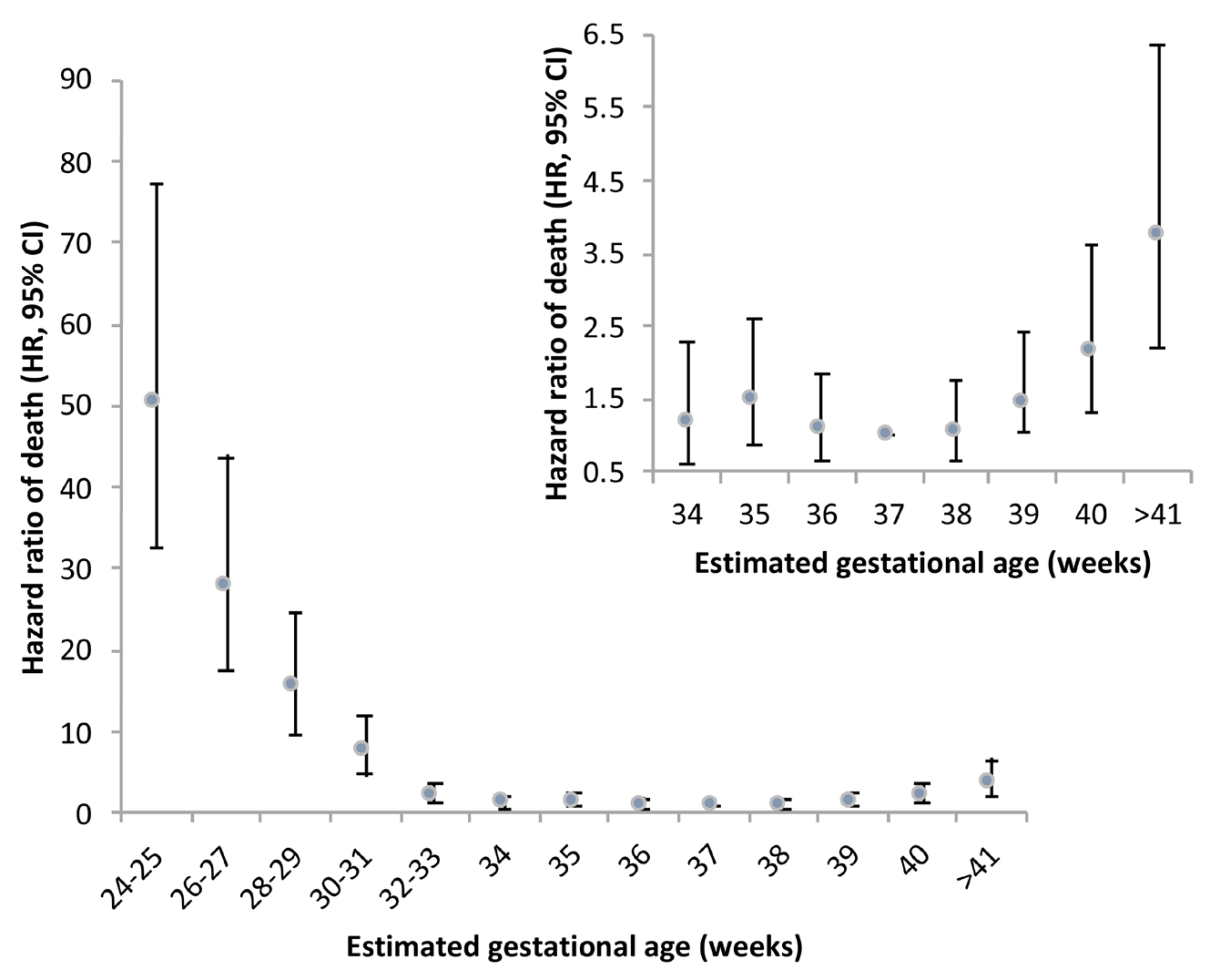
Hazard ratios of perinatal death in twins by gestational age at delivery.

Most perinatal deaths occurred in the extreme preterm period of 24–25 weeks (n = 99, 81.15%). Table 2 summarises the results of the univariate and multivariate Cox proportional hazards regression analyses using outcomes at 37–38 weeks as the referent. After adjusting for potential confounders, compared to delivery at 37–38 weeks, delivery at or above 39 weeks was associated with an increased risk of perinatal death (aHR 2.00, 95% CI 1.45–2.78). Delivery before 37 weeks was also associated with an increased risk of perinatal death (less than 32 weeks aHR 17.86, 95% CI 13.47-23.69, 33-36 weeks aHR 1.40, 95% CI 1.02-1.97). When the results were analysed by individual weeks, with 37 weeks as the referent, the relationship between perinatal death and gestation at delivery was reverse J-shaped ( Figure 2) with a decreasing risk of perinatal death with increasing gestational age up to 35 weeks. There was a very strong association between extreme preterm birth and perinatal death (24–25 wk: aHR 50.23 [95% CI 32.62-77.34], Figure 2). The results were similar when we ran the Cox regression analyses for the n= 5,269 twin infants with information available on maternal smoking (24–32 weeks compared to 37–38 weeks aHR 17.48 [95% CI 12.13-25.18], 33–36 weeks compared to 37–38 weeks aHR 1.16 [95% CI 0.71-1.75] and greater than 38 weeks compared to 37–38 weeks aHR, 3.10 [95% CI 2.07-4.77]).

**Table 2.** Univariate and Multivariate Cox regression analysis with robust standard errors of the association between gestational age at delivery and perinatal death in twin pregnancies (n=7,176).

Pregnancy Characteristic	N	Perinatal deaths N	Crude Hazard ratio (95% CI)	Adjusted hazard ratio (95% CI) ^#^
Gestational age (wks) 24–32 33–36 37–38 ≥39	740 2875 2363 1442	276 101 64 103	18.20 (13.87-23.89) 1.40 (1.03-1.92) 1 2.50 (1.83-3.41)	17.86 (13.47-23.69) 1.41 (1.01-1.97) 1 2.01 (1.45-2.78)
Maternal Age (yrs) 15–20 21–24 25–29 30–34 35–39 >40	486 1290 2272 2108 1066 198	45 126 161 127 72 13	1.34 (0.95-1.89) 1.42 (1.11-1.81) * 1 0.84 (0.66-1.07) 0.95 (0.71-1.27) 0.92 (0.51-1.65)	1.04 (0.72-1.52) 1.11 (0.87-1.41) 1 0.94 (0.74-1.20) 1.19 (0.90-1.58) 0.94 (0.54-1.63)
Maternal Height (cm) 141–150 151–160 161–170 >170	224 2706 3546 702	13 239 247 28	0.64 (0.36-1.13) 1 0.77 (0.64-0.93) * 0.43 (0.39-0.64) *	0.59 (0.35-1.01) 1 0.88 (0.74-1.06) 0.74 (0.49-1.34)
Year of delivery 1950–75 1976–2000 2001–2013	1957 3347 2116	220 207 117	2.16 (1.71-2.73) * 1.13 (0.89-1.42) 1	1.86 (1.38-2.52) * 1.02 (0.78-1.33) 1
Marital status Married Other	6034 1382	451 93	1 0.89 (0.71-1.23)	1 1.02 (0.81-1.30)
Parity Primigravida Parous	3077 4343	254 290	1.26 (1.06-1.50) * 1	1.09 (0.90-1.33) 1
Maternal complications No Yes	4377 3042	377 167	1 1.62 (1.34-1.96)	1 1.27 (1.05-1.54) *
Medical indication for induction No Yes	5730 1690	474 70	1 2.08 (1.62-2.68)	1 1.08 (0.81-1.44)

^#^adjusted for maternal age, height, year of delivery, marital status, parity, maternal complications, and medically indicated induction.

*Statistically significant at p<0.05

### Perinatal outcomes according to gestation at delivery stratified by chorionicity

**Figure 3.**
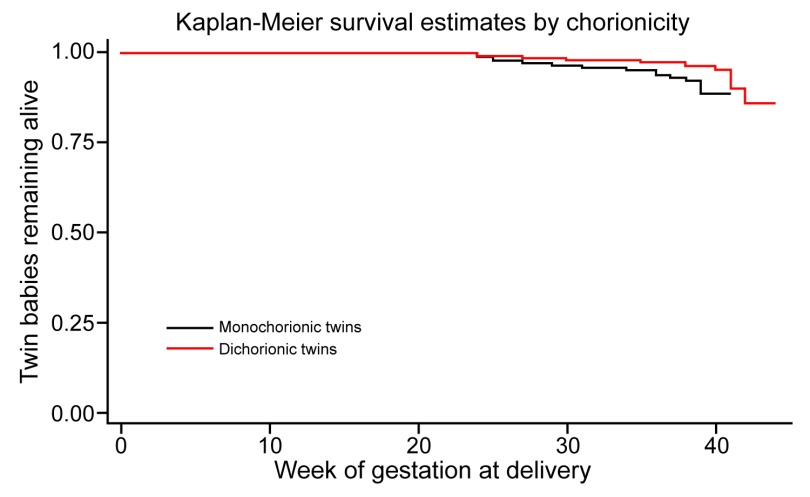
Kaplan-Meier plot of gestational age and perinatal death stratified by chorionicity.

Data on chorionicity was available for 5,130 twin babies, of which 4,134 (81%) were dichorionic and 996 (19%) were monochorionic ( Figure 1). There was a highly statistically significant difference in survival between monochorionic and dichorionic twins (overall HR for death in monochorionic twins compared to dichorionic twins 2.15, 95% CI 1.60-2.90), log rank test value 37.41, p<0.001, Figure 3).

In dichorionic twin pregnancies, compared to delivery at 37–38 weeks, only deliveries less than 32 weeks gestation had higher rates of perinatal death (aHR 30.14, 95% CI 17.94-50.64). Similarly, in monochorionic twin pregnancies delivery at less than 32 weeks was the group with a significantly higher risk of perinatal death than those delivered at 37–38 weeks (aHR 25.56, 95% CI 10.09-64.75).

### Perinatal outcomes according to gestation at delivery stratified by conception by assisted reproduction technologies

**Figure 4.**
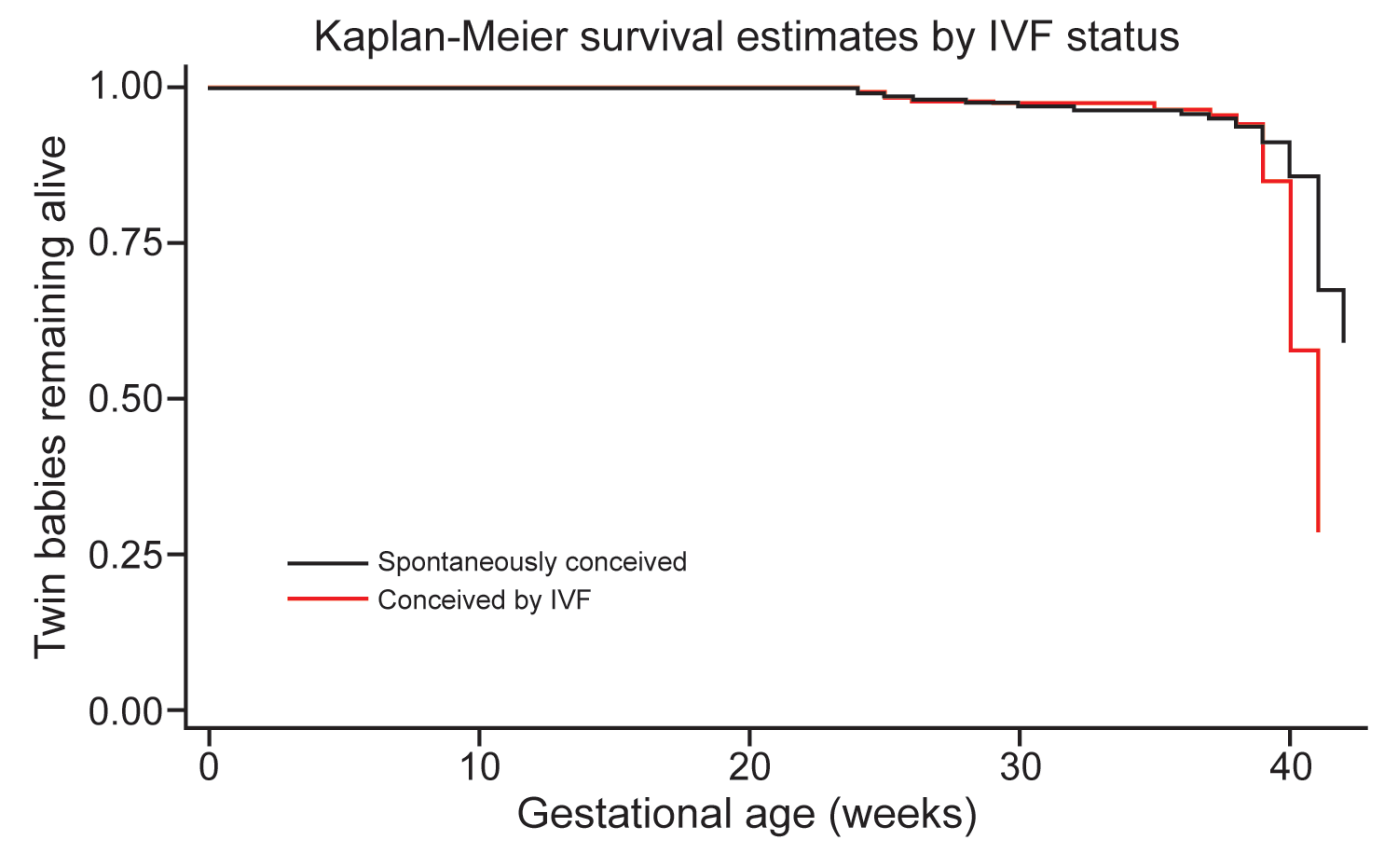
Kaplan-Meier plot of gestational age and perinatal death stratified by in vitro fertilization or spontaneous conception.

Data on ART conception was available on 5,523 twin infants, of which 722 (13.07%) were conceived by ART procedures. There was no evidence of a difference in survival between ART conceived and naturally conceived twins (overall HR for perinatal death in ART conceived twins compared to spontaneously conceived twins 1.09, 95% CI 0.79-1.50, log rank test value 3.64, p=0.07, Figure 4).

ART conceived twins were also no more likely to deliver preterm compared to spontaneously conceived twins (aHR 1.02, 95% CI 0.85-1.24). In both twins conceived by ART and those twin pregnancies spontaneously conceived compared to delivery at 37–38 weeks there was an increased risk of perinatal death in deliveries less than 32 weeks (aHR 18.58 [95% CI 12.70-27.19] in spontaneously conceived twins and aHR 19.91 [95% CI 6.54-60.71] in ART conceived twins) and in deliveries at or beyond 39 weeks (aHR 2.91, 95% CI 1.87-4.54 in spontaneously conceived twins and aHR 19.49, 95% CI 6.60-57.59 in ART conceived twins).

## Discussion

### Main findings

This study showed that the lowest rate of perinatal death for twins occurred in those delivered between 37 and 38 weeks gestation. Compared with this gestational age at delivery, there was almost a 2-fold increase in perinatal death in deliveries before 37 weeks, and a 2-fold increase in perinatal death in twin babies delivered at or beyond 39 weeks after adjusting for potential confounders. Although most of the results were presented in groups of gestational age weeks due to data sparsity, when the results were analysed by individual weeks of gestation, compared to delivery at 37 weeks, the differences in perinatal death were only statistically significant in deliveries before 35 weeks and above 39 weeks gestation. It is likely that the study was underpowered to show a difference in deaths between individual weeks of gestation because of the small number of perinatal deaths in the cohort and particularly in the later gestational weeks.

Guidance from NICE, UK on timing of delivery recommends elective delivery of twins from 37 weeks in dichorionic twins and 36 weeks in monochorionic twins in order to reduce perinatal death ^6^, and a subsequent systematic review upheld these recommendations ^10^. Our findings agree with delivery at 37–38 weeks to reduce perinatal death as per the national policy but we have not shown that delivery at 36 weeks is significantly different from delivery at 37 weeks in any of the groups. It is important to note however that our sample of monochorionic twins was likely too small to draw conclusions about individual gestational week categories, and although overall there was a 2-fold increase in perinatal deaths in monochorionic twins compared to dichorionic twins, from this study we are unable to relate this to gestational age at delivery. 

In contrast to some previous studies, we did not find any difference in perinatal death or preterm delivery in twins conceived by ART compared to those spontaneously conceived ^11, 14, 24^ and therefore this subgroup of twins should be managed according to the current UK guidelines. Taken together, this information is of use to clinicians planning the antenatal clinical management of twins and/or advising families with twin pregnancy.

### Strengths and limitations

The main strengths of this study are the large, unselected twin sample size in a stable population with high quality data. In particular, data on ART use and indication for induction of labour are rarely available. The retrospective cohort design allowed for efficient use of the routinely collected data. Another strength is the use of accurate gestational age measurements (we excluded pregnancies with inaccurate gestational age measurement) and the completeness of the dataset used. Inaccurate gestational age measurement is often a reason for variations in term and preterm rates between countries ^25^. Using routinely collected data ensured that every twin pregnancy was included thus reducing the risk of selection bias. We also accounted for the clustering effect of twins within mothers (and hence their similarity to each other) by estimating robust standard errors when producing the estimates and 95% confidence intervals. We believe the results of this study will be generalizable to the UK population.

There are of course some limitations to the use of routinely collected data. Missing covariate values can lead to selection bias if the missing values are not missing at random and can also result in a reduced sample size if included in multivariable analyses leading to a loss of power. In this study, we took the pragmatic approach of not including any covariates with large amounts of missing values but we did examine the effect of these variables in sensitivity analyses (which corroborated the findings) and we only used records with complete recordings for the stratified analyses. One of the caveats of using routinely collected data is that we are limited in the confounders adjusted for in the model to those that are routinely collected. A potential confounder we were unable to address was place of delivery (a potential confounder as women having a home birth are low risk and therefore different to those delivering in the hospital setting). However, given the small proportion of women who delivered outside of the AMH (99% of deliveries in Grampian are at the AMH which is the source of data collection for the AMND), we believe it is unlikely to have introduced any bias, especially with a twin pregnancy study where a home birth would be very unlikely in any geographical area. Another limitation is the omission of maternal weight in the multivariable analyses as a potential confounder, this decision was made due to the amount of missing values in the recording of this variable (>40%). The long period of time over which the study population was collected is another potential limitation of the study. Obstetric and neonatal care has likely changed over that time. We adjusted for this in the multivariable analysis by treating year of delivery as a possible confounder.

## Conclusions

In conclusion, perinatal death in twins appears to be lowest in twins delivered from week 37 and by the end of week 38. In keeping with previous studies, perinatal death was 2-fold higher in monochorionic twins compared to dichorionic twins, but we did not find any evidence in our study that they should be delivered at differing gestational ages, although the sample size for this subgroup was small. In contrast to some previous studies, we did not find any difference in perinatal mortality between twins born by ART procedures and twins spontaneously conceived and therefore twins conceived by ART should be managed according to the national guidelines. This information should be used when planning antenatal care and counselling women regarding optimum timing of delivery of twin pregnancies.

## Data availability

Data cannot be openly shared because it is sensitive human data. Access to the data can be requested through the AMND steering committee and individual study protocols are approved ( https://www.abdn.ac.uk/iahs/research/obsgynae/amnd/access.php).

Those wishing to gain access should complete a Databank request application form.

All applications and queries should be addressed to Dr. Sohinee Bhattacharya ( sohinee.bhattacharya@abdn.ac.uk)
