# Supplementary material for: Gestational age at delivery of twins and perinatal outcomes: a cohort study in Aberdeen, Scotland
Source: Wellcome Open Res. 2019 Jul 22;4:65. Originally published 2019 Apr 3. [Version 2] doi: 10.12688/wellcomeopenres.15211.2 (PMC6688720; doi:10.12688/wellcomeopenres.15211.2)
Supplement: Peer review history

# Peer review: Reviewer response for version 2

**DOI:** 10.21956/wellcomeopenres.16783.r36019

**Published:** 2019-08-07

**Authors:** Sebastian Manzanares

**Affiliations:** 1 Obstetrics and Gynecology Department, Virgen de las Nieves University Hospital, Granada, Spain

All previous comments have been included to the new version. So the paper is approved.

I confirm that I have read this submission and believe that I have an appropriate level of expertise to confirm that it is of an acceptable scientific standard.

# Peer review: Reviewer response for version 1

**DOI:** 10.21956/wellcomeopenres.16599.r35463

**Published:** 2019-06-21

**Authors:** Nigel A.B. Simpson

**Affiliations:** 1 Department of Obstetrics and Gynaecology, University of Leeds, Leeds, UK

This may not be the most original study, and the findings are not at variance with those of recent studies and recommendations, but there is a pleasing exactitude and approach in the way in which the authors have sought to examine the relationship between twin mortality and gestational age within a geographically well-demarcated, robustly-curated dataset from Northern Scotland which is well-written, easy to follow, and does not pretend to present anything else other than the affirmation of pre-existing wisdom on the subject. I would strongly endorse its indexing as being of interest to the site's readership.

I confirm that I have read this submission and believe that I have an appropriate level of expertise to confirm that it is of an acceptable scientific standard.

# Peer review: Reviewer response for version 1

**DOI:** 10.21956/wellcomeopenres.16599.r35385

**Published:** 2019-05-08

**Authors:** Sebastian Manzanares

**Affiliations:** 1 Obstetrics and Gynecology Department, Virgen de las Nieves University Hospital, Granada, Spain

It's a quite large and interesting retrospective study that concludes that delivery of twins at 37-38 weeks gestation is associated with the lowest perinatal mortality rate.

 Some comments to authors:

- In the Abstract, the result that indicate that there was almost a 2-fold increase in perinatal death in deliveries before 37 weeks should be included.
- In the Keywords, twin pregnancy should be added. 
- In the Introduction, when you assert that twin birth rate has risen and is set to continue to rise, you should provide a reference to support this.
- In the Methods, maternal body mass index or at least maternal weight should be included as a variable, provided that it's available. Birth weight is also an important variable to consider in mortality studies.

I confirm that I have read this submission and believe that I have an appropriate level of expertise to confirm that it is of an acceptable scientific standard, however I have significant reservations, as outlined above.

# Response

**Published:** 2019-07-11

**Authors:** Sarah Murray

**Affiliations:** University of Edinburgh, UK

**​​​​​​Author Response to Reviewer**

Thank you very much for your review - I have made the suggested changes and they can now be reviewed in version 2 of the manuscript. The changes are detailed below:

*In the Abstract, the result that indicate that there was almost a 2-fold increase in perinatal death in deliveries before 37 weeks should be included.*

This is now included in the abstract.

*In the Keywords, twin pregnancy should be added.*

This has now been included.

*In the Introduction, when you assert that twin birth rate has risen and is set to continue to rise, you should provide a reference to support this.*

A reference has been added for this statement.

*In the Methods, maternal body mass index or at least maternal weight should be included as a variable, provided that it's available. Birth weight is also an important variable to consider in mortality studies.*

I have now included a section in the limitations of the study detailing why maternal weight was not included in the multivariate model.
